# Comprehensive analysis of microRNA expression in regionalized human neural progenitor cells reveals microRNA-10 as a caudalizing factor

**DOI:** 10.1242/dev.122747

**Published:** 2015-09-15

**Authors:** Marie E. Jönsson, Jenny Nelander Wahlestedt, Malin Åkerblom, Agnete Kirkeby, Josephine Malmevik, Per Ludvik Brattaas, Johan Jakobsson, Malin Parmar

**Affiliations:** 1 Lab of Molecular Neurogenetics, Wallenberg Neuroscience Center and Lund Stem Cell Center, Lund University, Lund 221 84, Sweden; 2 Developmental and Regenerative Neurobiology, Department of Experimental Medical Science, Wallenberg Neuroscience Center and Lund Stem Cell Center, Lund University, Lund 221 84, Sweden

**Keywords:** Cell specification, Neural development, Neural stem cells, MicroRNA

## Abstract

MicroRNAs (miRNAs) have been implicated in regulating multiple processes during brain development in various species. However, the function of miRNAs in human brain development remains largely unexplored. Here, we provide a comprehensive analysis of miRNA expression of regionalized neural progenitor cells derived from human embryonic stem cells and human foetal brain. We found miR-92b-3p and miR-130b-5p to be specifically associated with neural progenitors and several miRNAs that display both age-specific and region-specific expression patterns. Among these miRNAs, we identified miR-10 to be specifically expressed in the human hindbrain and spinal cord, while being absent from rostral regions. We found that miR-10 regulates a large number of genes enriched for functions including transcription, actin cytoskeleton and ephrin receptor signalling. When overexpressed, miR-10 influences caudalization of human neural progenitor cells. Together, these data confirm a role for miRNAs in establishing different human neural progenitor populations. This dataset also provides a comprehensive resource for future studies investigating the functional role of different miRNAs in human brain development.

## Abstract (precis)

**Summary:** The profiling of neural progenitors derived from human ESCs and foetal brain shows that miRNAs display region-specific expression patterns, suggesting that they contribute to establishing regional identity.

## INTRODUCTION

MicroRNAs (miRNAs) are small noncoding RNAs that mediate post-transcriptional regulation of gene expression (Bartel, 2004). A single miRNA can have up to several hundreds of target genes, and, so far, more than a thousand miRNAs have been discovered in the human genome (Kozomara and Griffiths-Jones, 2014), suggesting that these molecules are important players in controlling transcriptional networks (Guo et al., 2010; Hafner et al., 2010; Helwak et al., 2013). An increasing body of evidence in model organisms and human cells suggests that miRNAs have an important role in neural determination, differentiation and cell fate specification (Delaloy et al., 2010; Du et al., 2013; Fineberg et al., 2009; Makeyev et al., 2007; Smrt et al., 2010; Stappert et al., 2013). However, studies on the role of miRNAs during human brain development are hampered by the lack of human foetal tissue and the difficulties in obtaining purified populations of neural progenitors of a defined rostro-caudal identity. To date, only a few miRNAs have been demonstrated to play a role in human neural differentiation and development (Du et al., 2013; Roese-Koerner et al., 2013; Stappert et al., 2013; Sun et al., 2013), and little is known about their potential regional expression and possible involvement in subtype-specific neuronal differentiation.

Recent advances in the differentiation protocols of human pluripotent stem cells (hPSCs) that recapitulate human neural development, and which produce neural progenitors and neurons very similar to the *in vivo* counterparts, have made it possible to model human brain development using hPSCs. This is advantageous, as it offers an unlimited availability of regionalized human neural progenitors, and also because it allows for genetic modifications and selection of the cells. We have recently established a defined protocol for human embryonic stem cell (hESC) differentiation that mimics early human neural development. In this protocol, precisely dosed chemical activation of canonical Wnt signalling is combined with SHH to yield authentic, regionalized neural progenitors and neurons that are very similar to their *in vivo* counterparts (Grealish et al., 2014; Kirkeby et al., 2012a,b).

In this study, we generated a *SOX1-*GFP reporter cell line in order to monitor and purify human neural progenitors in live cultures. Using this line and the cell-surface marker CORIN for floor-plate (FP) cells we obtained pure populations of neural progenitor and FP cells of a forebrain (FB), midbrain (MB) and hindbrain (HB) identity, and we performed global miRNA expression profiling of regionalized human progenitors. We also performed a miRNA array on human foetal samples to confirm and complement our sequencing data. This comprehensive analysis provides a miRNA profile of neural progenitors from different regions of the human brain and shows that miRNAs display region-specific expression patterns in human neural progenitors. We identified miR-10 to be highly expressed specifically in the HB and spinal cord (SC), and identified 89 high-confidence miR-10 target genes, enriched for functions related to transcription, actin cytoskeleton and ephrin receptor signalling. Finally, gain-of-function experiments suggested a key role for miR-10 in caudalizing human neural progenitors (hNPCs).

## RESULTS

### Generation of a *SOX1*-GFP hESC reporter cell line

The transcription factor Sox1 is expressed by neural progenitors cells throughout the neuroepithelium (NE), and has previously been used as a reporter gene to monitor early neural fate commitment in mice and in differentiating mouse embryonic stem cells (mESCs) (Aubert et al., 2003; Kan et al., 2004; Lang and Shi, 2012; Venere et al., 2012). However, in the mouse MB, some ventral neural progenitors are FP cells and do not express Sox1. These progenitors can instead be identified based on expression of the FP-specific cell-surface marker Corin (Kirkeby et al., 2012a; Ono et al., 2007). To determine whether Sox1 and Corin could potentially be used to isolate pure NE and FP populations of neural progenitors from a human origin, we performed an expression analysis on human foetal brains and found that both SOX1 and CORIN are expressed in the same regions in humans as in rodents (supplementary material Fig. S1).

**Fig. 1.**
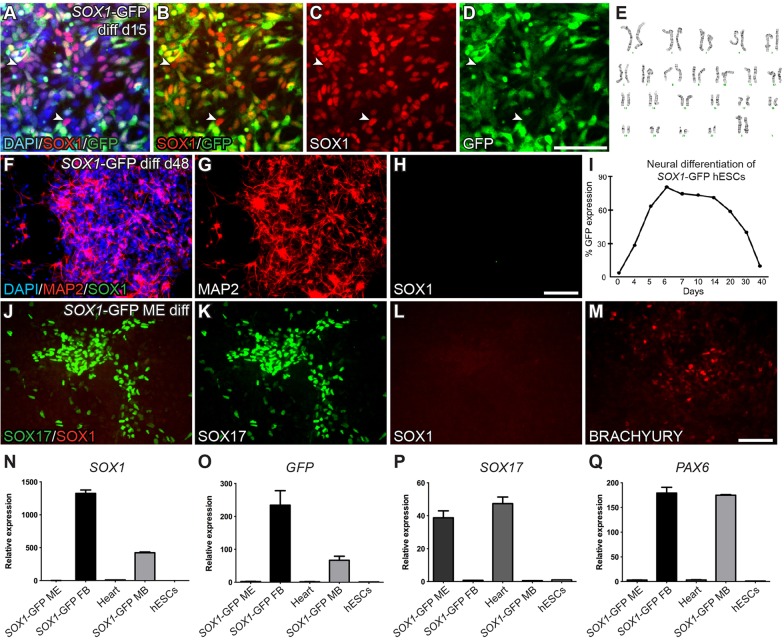
**The clonal *SOX1*-GFP hESC line monitors differentiation of neural epithelial cells.** (A-D) Upon neural induction, the clonal cell line *SOX1*-GFP rapidly switched on GFP, which overlapped with SOX1 expression. Only a very small fraction of the SOX1^+^ cells were GFP^−^ (<2%, indicated by white arrowheads). (E) The *SOX1*-GFP cells retained a normal karyotype (46,xx). (F-H) As the cells were terminally differentiated, MAP2^+^ neurons no longer expressed SOX1. (I) The GFP expression was monitored during differentiation using FACS. GFP expression peaked around day 6 and was maintained at high levels until terminal differentiation was initiated at day 16. (J-M) Upon mesendodermal (ME) differentiation, no SOX1 was detected. Instead, cells started to express the ME markers SOX17 and BRACHYURY. (N-Q) The expression of *SOX17* as well as the absence of *GFP* and *SOX1* was confirmed with qRT-PCR. ME cells were also found negative for the neural marker *PAX6*. All qRT-PCR analyses were performed using three biological and three technical replicates. Data are presented as mean±s.e.m. Scale bars: 100 μm.

As CORIN is a cell-surface marker, it is possible to purify the cells using antibodies and fluorescence-activated cell sorting (FACS). For isolation based on *SOX1* expression, we generated a hESC reporter cell line expressing GFP under the control of *SOX1* regulatory sequences (*SOX1*-GFP) using BAC recombineering (supplementary material Fig. S2). The BAC-based *SOX1*-GFP reporter construct (supplementary material Fig. S2A) was introduced into hESCs by nucleofection, followed by clonal expansion and differentiation (supplementary material Fig. S2B). We identified one clone that robustly initiated GFP expression in SOX1^+^ cells upon neuralization (Fig. 1A-D), and we confirmed that this clone retained a normal karyotype (46,xx) (Fig. 1E). There was an almost complete overlap (>98%) between GFP and SOX1 when analysed 15 days after initiation of neuralization (Fig. 1A-D). When analysing the GFP expression upon neuronal differentiation and maturation, we confirmed that SOX1 and GFP were downregulated upon terminal differentiation into mature neurons (Fig. 1F-H,I). To rule out the possibility that GFP reported a general differentiation event, we differentiated the hESC *SOX1-*GFP reporter cells to mesendodermal (ME) cells according to a published protocol (D'Amour et al., 2006). After 7 days, clusters of cells were found positive for the ME markers SOX17 (Fig. 1J,K) and BRACHYURY (coded by the gene *T* – HUGO Gene Nomenclature Committee) (Fig. 1M), and no SOX1 or GFP expression was observed (Fig. 1J,L). In addition, quantitative real-time PCR (qRT-PCR) analysis showed a high expression of *SOX17* compared with controls, and no expression of *SOX1*, *GFP* or the neural progenitor marker *PAX6* could be detected (Fig. 1N-Q). Thus, we confirmed that the *SOX1*-GFP reporter cell line reliably reports *SOX1* expression in human neural progenitor cells, whereas GFP expression is absent from differentiated neurons, undifferentiated hESCs and cells of non-ectodermal lineages.

### Isolation of purified regionalized human neural progenitor cells

**Fig. 2.**
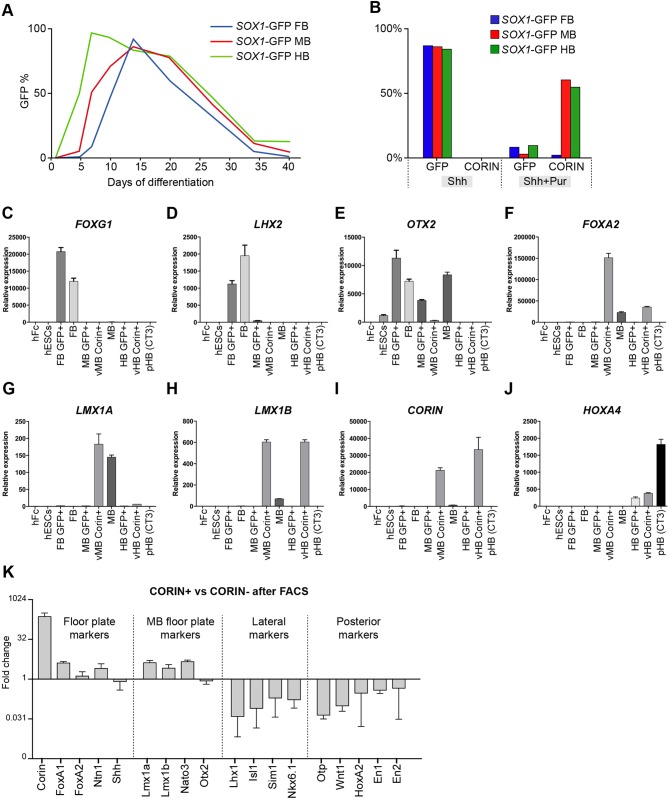
**Isolation of purified regionalized human neural progenitor cells.** (A) The optimal time point for isolation of early patterned neural progenitors was decided by monitoring the expression of GFP by FACS upon neural differentiation for 40 days. The GFP expression in FB and MB NE cells peaked at day 14, whereas the GFP expression of HB NE peaked already at ∼day 6 and stayed high until terminal differentiation was initiated at day 16. (B) Cells were analysed by FACS at day 14 of differentiation. The vast majority of NE cells express GFP, whereas the FP marker CORIN is absent. In order to ventralize the cells effectively, the SHH agonist Purmorphamine (Pur) was added to the media, in addition to SHH, during day 0-9 of differentiation, resulting in the majority of cells being GFP^–^ at day 14, and FP cells from MB and HB cultures starting to express high levels of CORIN. (C-J) Regionalization of *SOX1*-GFP ESCs patterned towards FB, MB or HB was investigated at day 14 using qRT-PCR. FB patterning was analysed by the expression of *FOXG1*, *LXH2* and *OTX2*. MB patterning was analysed by the expression of *OTX2*, *FOXA2* and *LMX1A/B*. HB patterning was analysed by the expression of *HOXA4*. The ability to form FP cells was analysed by the expression of *CORIN*. (K) Isolation of the CORIN^+^ population from cultures patterned towards ventral MB resulted in enrichment of FP and mesDA markers, whereas cells expressing lateral and posterior markers are excluded. All qRT-PCR analyses were performed using three biological and three technical replicates. Data are presented as mean±s.e.m.

To define the optimal time point for isolation of early *SOX1*^+^ neural progenitors, we differentiated the cells using dual SMAD inhibition for neuralization (Chambers et al., 2009), and SHH and chemical WNT activation for dorso-ventral and rostro-caudal regionalization as described (Grealish et al., 2014; Kirkeby et al., 2012a,b). We analysed the proportion of GFP expression upon differentiation to FB, MB and HB over time in culture. We observed a rapid increase of GFP expression in all three conditions upon neural differentiation, and a decline in expression as the cells differentiated towards mature neurons (Fig. 2A). The FB and MB cells peaked in GFP expression around day 14 of differentiation, whereas in the cells in the HB condition GFP expression was shown to peak already at day 6 and stayed at high levels until terminal differentiation was initiated from ∼day 16 (Fig. 2A). Based on these data, we decided to isolate progenitors from all three conditions at day 14 of differentiation. The differentiations into FB, MB and HB were monitored using qRT-PCR, and we confirmed the enrichment of the FACS-isolated populations with a selection of markers previously used to characterize regionalization of differentiated hESCs (Fig. 2C-J) (Kirkeby et al., 2012b). To obtain enriched populations of MB FP progenitors (which do not express SOX1), we added the SHH agonist Purmorphamine (Pur) to the cultures from day 0 to 9 for efficient ventralization of the cells (Kriks et al., 2011). In the presence of Pur, the expression of GFP was heavily decreased in all three conditions (Fig. 2B; supplementary material Fig. S3A-F), and, within the MB and HB cultures, a significant proportion of the cells were identified as CORIN^+^ and FOXA2^+^ (supplementary material Fig. S3J-Q), consistent with a floor-plate structure in these regions of the embryo (Shimamura et al., 1995). Using qRT-PCR we confirmed that the FACS-sorted CORIN^+^ population was enriched in MB FP markers (Fig. 2K).

Thus, upon FACS isolation of the GFP^+^ cells from FB, MB and HB, and the GFP^−^/CORIN^+^ cells from ventralized MB and HB conditions, we were able to obtain pure populations of NE and FP neural progenitors to be used for sequencing.

### miRNA profiling of early human neural progenitors

**Fig. 3.**
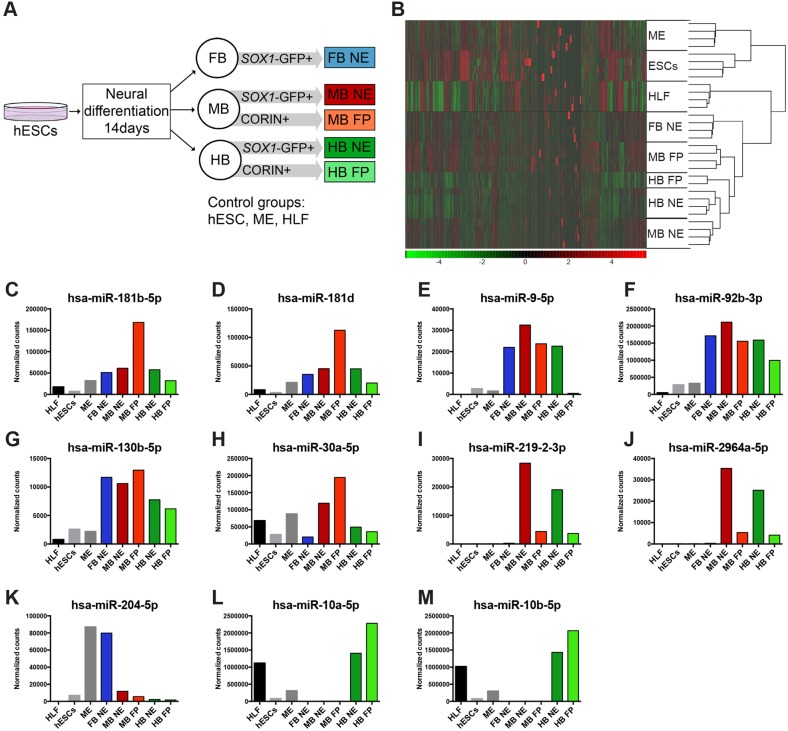
**Expression of miRNAs in hESC-derived neural progenitors.** (A) The different groups of cells were generated by differentiating *SOX1*-GFP hESCs towards FB, MB or HB for 14 days. At that point, cells were sorted for GFP^+^ or CORIN^+^, generating the NE and FP groups, respectively. (B) Heat map showing a random clustering analysis in which each neural subtype clustered together, revealing that cells possess a specific global miRNA expression pattern. (C-M) miRNAs with a fourfold or higher expression in NE cells compared with hESCs were extracted, and the normalized counts for selected miRNAs are shown.

miRNAs have a large impact on the transcriptome and have been shown to control neural fate determination and cell specification (Akerblom and Jakobsson, 2014). To elucidate the role of miRNAs in early human lineage specification, we performed deep sequencing of small RNA isolated from the purified neural populations. Samples included in this experiment were sorted from *SOX1-*GFP hESCs differentiated towards NE cells of FB, MB and HB, as well as FP cells from MB and HB (Fig. 3A). As control cells we used undifferentiated hESCs, human lung fibroblasts (HLF) and ME cells (Fig. 3A). The sequencing reads were mapped to miRBase, normalized and subsequently analysed for the expression levels for different miRNAs (supplementary material Table S1).

Unbiased clustering of the data demonstrated that the different neural progenitor subgroups clustered together, separating these populations from cells of the non-ectodermal lineages, ME cells, HLFs and hESCs (Fig. 3B). In addition, each neural subtype clustered together (Fig. 3B). Thus, these data demonstrated that the miRNA expression can be used to distinguish human neural progenitors of different subtypes and suggest that miRNAs play a role in establishing different human neuronal subtypes.

To identify miRNAs with a possible role in early neuronal lineage selection and fate-specific differentiation during hESC differentiation, we selected individual miRNAs that had a fourfold or higher expression in FB, MB and/or HB NE cells compared with hESCs. For this analysis, we omitted the HB FP samples, as the HB FP has been shown to be non-neurogenic in the mouse. We identified 30 miRNAs that were considerably enriched in human neural progenitors, and a large proportion of these were expressed at low levels in non-ectodermal cells (Table 1). The highest-expressed miRNAs in FB, MB and/or HB NE cells were extracted for closer analysis (Fig. 3C-M).

**Table 1.**
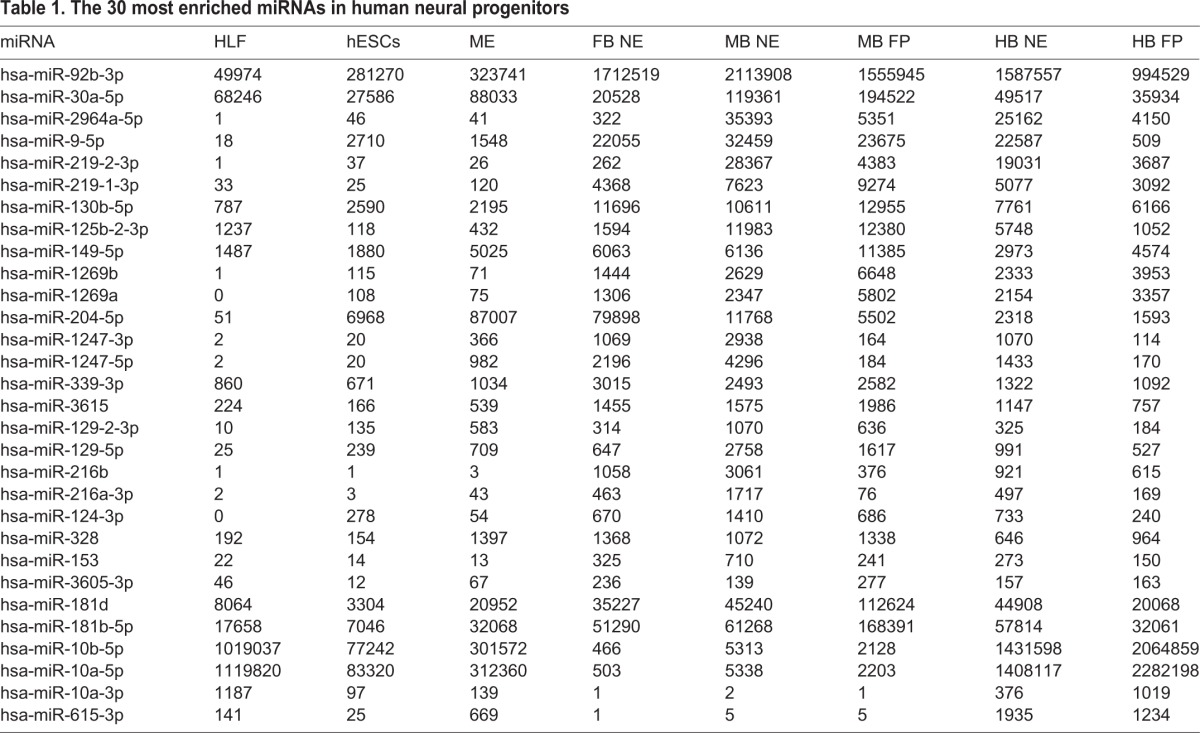
**The 30 most enriched miRNAs in human neural progenitors**

We found that members of the miR-181 family and miR-9-5p were highly expressed and considerably enriched in human neural progenitors (Fig. 3C-E), which is in line with other studies (Stappert et al., 2013; Yoo et al., 2011). MiR-92b-3p and miR-130b-5p were identified as two miRNAs highly expressed in FB, MB and HB cells compared with hESCs, and also compared with ME and HLFs (Fig. 3F,G). These two miRNAs are members of the miR-25 and miR-130 families, respectively, which previously have been identified for regulating cell proliferation (Lee et al., 2011; Xu et al., 2013). Moreover, miR-92b-3p and miR-130b-5p were the only members of their respective families to be specifically associated with neural progenitors (Fig. 3F,G), which suggests that they play a role in regulating human neural progenitor proliferation.

MiR-30a-5p, previously shown to target Smoothened to regulate Hedgehog signalling in zebrafish, was highly expressed in all groups of NE cells, although noticeably higher in MB cells (Fig. 3H, Table 1) (Ketley et al., 2013). MiR-219-2-3p and miR-2964a-5p were found specifically enriched in MB and HB NE cells (Fig. 3I,J). MiR-204-5p was associated with FB NE cells (Fig. 3K, Table 1). In mouse eye development, miR-204 has been linked to the transcription factor Pax6, as Pax6 directly upregulates miR-204 in order to repress multiple genes, such as *Sox2*, *Sox9* and *Sox11* (Conte et al., 2010; Shaham et al., 2013). Finally, two members of the miR-10 family, implicated both in brain development and in cancer development (Lund, 2010; Woltering and Durston, 2008), were highly expressed by and exclusively associated with HB cells (Fig. 3L,M, Table 1).

**Fig. 4.**
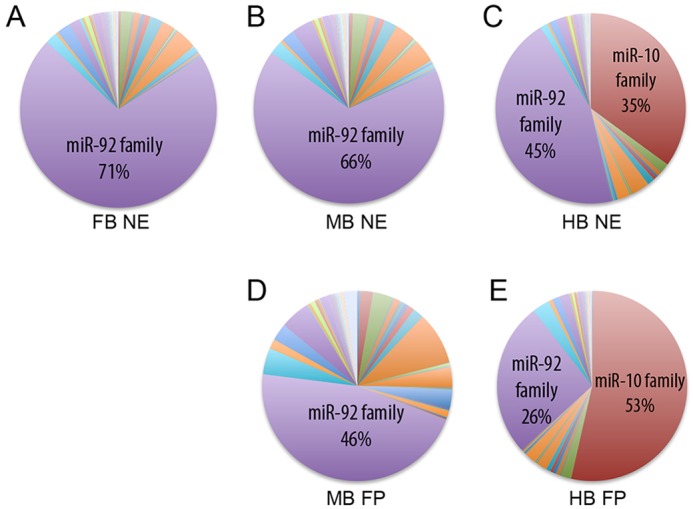
**Expression of miRNA families in human NE and FP cells.** (A-C) Circular charts demonstrating miRNA expression grouped into families. The miR-92 family constitutes a large proportion of all miRNA families expressed in NE cells patterned towards FB, MB and HB. In the HB NE cells, the miR-10-family represents 35% of all miRNAs, while it is absent from the FB and MB NE cells. (D,E) The proportion of miR-92 family expression is also high in FP cells from both MB and HB. Expression of the miR-10 family constitutes more than half of all miRNAs in the FP cells of the HB.

Next, we grouped the miRNAs into families and analysed their relative contribution to the total pool of miRNAs (Fig. 4; supplementary material Table S2). This analysis revealed that the miR-92 family dominates FB, MB NE and MB FP cells, making up a large proportion of all miRNA reads (Fig. 4A,B,D). However, HB NE cells display a large fraction of reads (35%) mapping to the miR-10 family (Fig. 4C). Similar enrichment in miR-10 family expression was also found in HB FP cells (Fig. 4E). These reads in the miR-10 family, which primarily maps to miR-10a and miR-10b, suggest that miR-10 family members have a unique spatial regulation, resulting in very high-level expression only in the hindbrain.

### Profiling of miRNA expression in human foetal brain cells

**Fig. 5.**
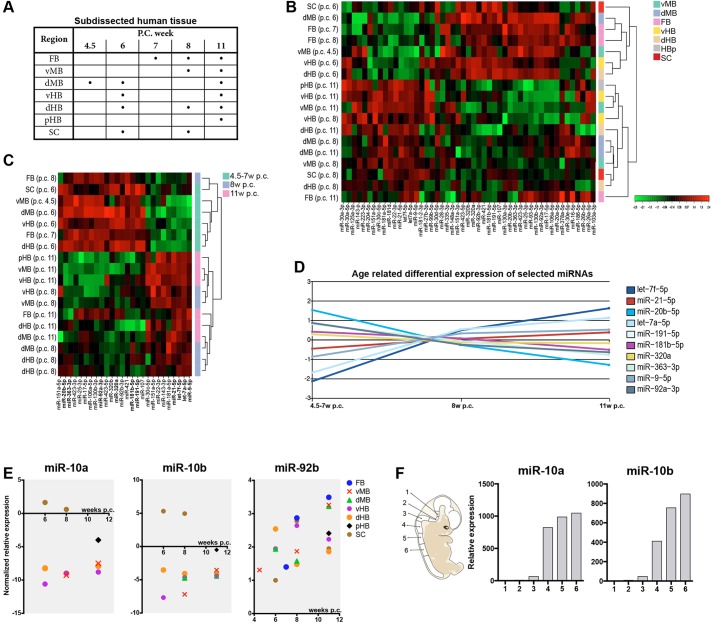
**Validation of miRNAs in human foetal brain development.** (A) Different dissected regions and ages from human foetal tissue used for the miRNA microarray. Each dot represents *n*=1. (B) Heat map showing an unbiased clustering analysis of samples using normalized dCq values. The clustering was performed on all samples, and on the top 49 microRNAs with the highest s.d. The colour scale illustrates the relative expression levels of microRNA across all samples. (C) Heat map showing scaled expression of miRNAs displaying large-magnitude changes significantly different between the time points. Colour scale as in B. (D) In order to visualize age-related expression patterns in the top 10 miRNAs from C, their centred average dCq-values were plotted to the three age groups. (E) miR-10a and miR-10b were only detected in the tissue obtained from the pHB and SC. Normalized, centred dCq-values from each sample were plotted by region and age. miR-92b was detected at high levels in all samples. (F) The expression of miR-10a/b was validated in sub-dissected HB to SC from a human foetus 8 weeks p.c. by LNA-RT-qPCR. FB, forebrain; vMB, ventral midbrain; dMB, dorsal midbrain; vHB, ventral hindbrain; dHB, dorsal hindbrain; pHB, posterior hindbrain; SC, spinal cord.

The miRNA-seq data show that different developing human brain regions can be segregated based on their miRNA-expression profile. To confirm that the data obtained from purified hESC-derived neural progenitors are relevant for actual human foetal brain development, we sub-dissected and collected regions from corresponding rostro-caudal levels of the developing neural tube from human foetuses of developmental stages spanning from onset of neurogenesis to peak production of neurons (Fig. 5A). We processed the material using the same small-RNA extraction kit as for the hESC-derived NPCs (hNPCs), and analysed the material using a custom-made microRNA array including 59 miRNAs, selected based on their expression pattern in the regionalized hNPCs.

First, this analysis confirmed that the great majority of miRNAs detected by miRNA-seq in hESC-derived progenitors could also be detected using qRT-PCR in the human foetal brain of corresponding regions. As the cell composition of the sub-dissected regions of the human foetal brain is more diverse than in the sorted hESC-derived progenitors, and as it is not possible to match exactly the developmental stages of the cells, a closer comparison between experiments is not possible. However, unbiased analysis of miRNA expression pattern in samples derived from human foetuses also confirmed independently that distinct expression profiles for different brain regions exist, including many of the regional expression patterns identified in the miRNA-seq experiment (Fig. 5B; supplementary material Table S3).

As we included samples from different developmental stages in this experiment, we were also able to analyse the impact of developmental stage on miRNA expression (Fig. 5C). In this analysis, we identified several miRNAs that display a stage-dependent expression, including miRNAs that are both upregulated and downregulated as development proceeds (Fig. 5D). Examples of miRNAs with increased expression as development proceeds include: let-7f, miR-9 and miR-21, whereas, for example, miR-20b and miR-363 display reduced expression with increasing embryo age (Fig. 5D).

### miR-10 display a robust regional-specific expression in the developing human brain

Next, we focused our analysis on miR-10, as this was the miRNA with the most distinct expression profile in the RNA-seq experiment, being highly expressed in HB but absent from FB and MB patterned human neural progenitors. We used the data from the miRNA array of developing human brain samples to verify the HB-specific expression of miR-10. We found that miR-10a and miR-10b displayed high levels of expression in the posterior regions, particular the SC, at all developmental time points analysed, while we were unable to detect miR-10 expression in any FB or MB samples (Fig. 5E), confirming the RNAseq data. Also in line with the RNA-seq data, we found that miR-92b was highly expressed in all brain regions and time points analysed (Fig. 5E).

We then extended the miR-10 analysis to also include qRT-PCR analysis of tissue samples dissected, including more caudal levels of the neuroaxis, and found that miR-10a and miR-10b expression is gradually increased in more posterior samples, reaching high levels in the SC (Fig. 5F). In summary, this expression profiling demonstrated that miR-10a and miR-10b are absent from FB and MB regions of the human developing neural tube. Expression is then initiated in the HB and gradually increases to reach high levels in the developing SC. Thus, miR-10 displays a highly specific regionalized expression pattern in the developing human brain, suggesting a role for miR-10 in caudalization of hNPCs.

### Overexpression of miR-10 caudalizes midbrain-patterned human neural progenitors

**Fig. 6.**
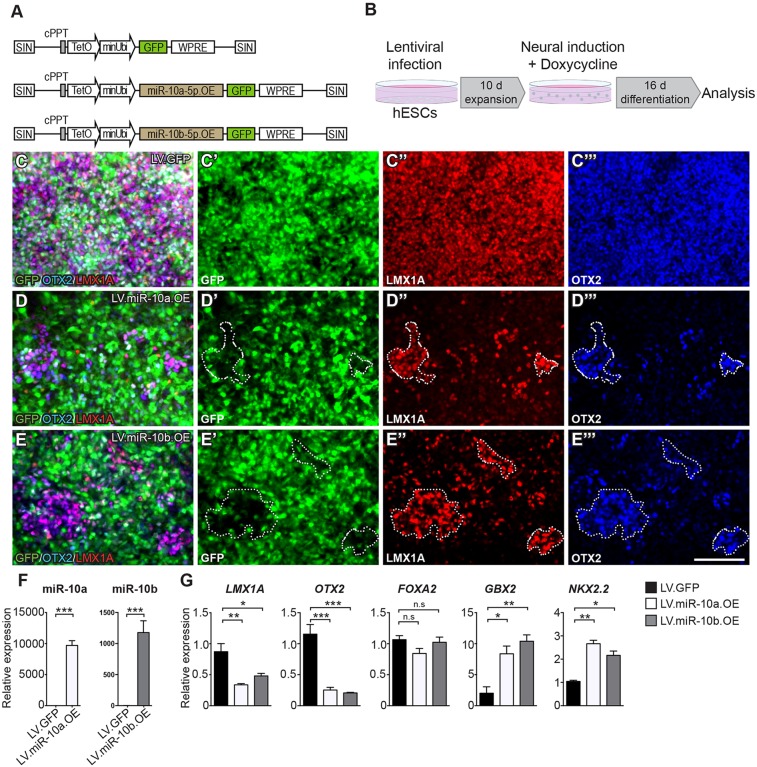
**Overexpression of miR-10a and miR-10b caudalizes MB-patterned human neural progenitors.** (A) Illustration of the proviral form of LV.GFP, LV.miR-10a.GFP and LV.miR-10b.GFP. (B) Schematic overview of the workflow showing that hESCs were transduced with lentiviral vectors followed by 10 days of expansion. Doxycycline was added to the media during differentiation and cells were analysed at day 16. (C-E‴) OTX2 and LMX1A are expressed in the vast majority of cells infected with LV.GFP (C-C‴), but only in a small proportion of the cells in which miR-10a or miR-10b are overexpressed (D-E‴). (F) The miR-10a and miR-10b expression was investigated using LNA-RT-qPCR at day 16 of differentiation, showing that miR-10a and miR-10b are strongly expressed in cells infected with LV.miR-10a.GFP and LV.miR-10b.GFP, respectively. (G) qRT-PCR of cells after 16 days of MB patterning revealed that cells overexpressing miR-10a or miR-10b had been caudalized in comparison to control cells. The MB markers *LMX1A* and *OTX2* decreased while the expression of the anterior HB marker *GBX2* increased. The expression of *NKX2.2* was also increased. Expression levels of *FOXA2* were unaffected, which correlates with the fact that *FOXA2* is expressed in both MB and HB during human development. All qRT-PCR analyses were performed using three biological and three technical replicates. Data are presented as mean±s.e.m. *P<0.05, **P<0.005, ***P<0.0005. Scale bar: 100 μm.

To investigate whether miR-10a/b has a functional role in human neural patterning, we generated lentiviral vectors that allow for doxycycline-regulated overexpression of miR-10a and miR-10b as well as a GFP-reporter. We also included a GFP-only vector as control (Fig. 6A). Transduction of hESCs with these vectors at a multiplicity-of-infection (MOI) of 50 resulted in robust expression of miR-10a/b expression upon addition of doxycycline (Fig. 6B-E). To investigate a role for miR-10 in caudalization of human neural progenitors, we transduced hESCs with the lentiviral miR-10a or miR-10b vectors as well as the GFP control vector and differentiated these cells towards a ventral MB identity. Doxycycline was added at the start of differentiation, resulting in induction of miR-10a/b expression (Fig. 6F); cells were then analysed using immunocytochemistry and qRT-PCR at day 16 of differentiation. A large fraction of the cells expressed GFP (Fig. 6C-E). We found that human MB cultures overexpressing miR-10a or miR-10b displayed reduced gene and protein expression of the ventral MB markers LMX1A and OTX2 (Fig. 6C-E,G), whereas *NKX2.2* and *GBX2*, which is expressed directly caudal of the MB-HB organizer during human neural development, were upregulated (Fig. 6G). Remarkably, we observed by immunocytochemistry that expression of the FB/MB marker OTX2 was suppressed in the vast majority of GFP^+^ miR-10-transduced progenitors, whereas patches of OTX2^+^ progenitors were largely restricted to colonies of non-transduced progenitors (Fig. 6D,E). By contrast, the expression of FOXA2, which is expressed in ventral MB as well as in ventral HB, was unaffected by overexpression of miR-10a/b, indicating that miR-10 did not affect the dorso-ventral patterning of the cells (Fig. 6G). Thus, we conclude that miR-10a/b overexpression results in caudalization of differentiating hNPCs.

### Identification of miR-10 target genes

**Fig. 7.**
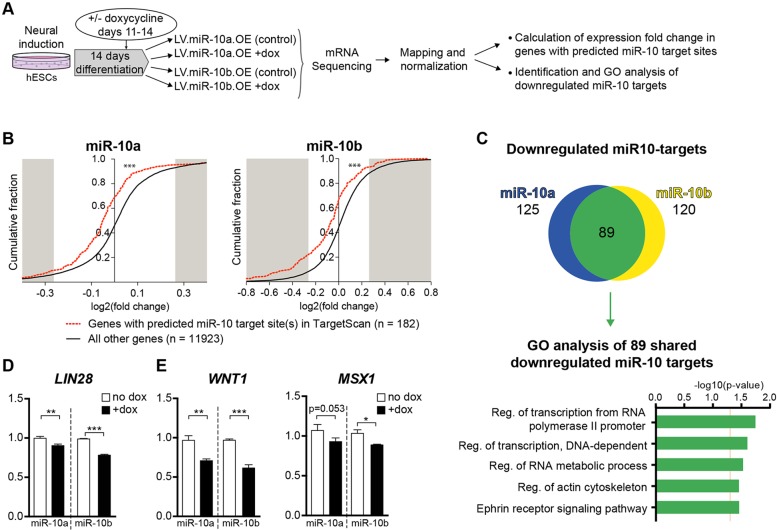
**Identification of miR10 targets in hNPCs.** (A) Schematic overview of the experiment. Transduced hESCs were differentiated towards ventral MB for 14 days. The expression of miR-10 was induced by adding doxycycline to the media on days 11-14, whereas no doxycycline was added to the control groups. All groups include three replicates. (B) The fold change (FC) of each gene after miR-10a or miR-10b overexpression was plotted in a cumulative fraction graph. The cumulative distribution of the FC values for predicted miR-10 targets (red dotted lines; *n*=182) was significantly lower (shifted to the left) in comparison to that of all other genes, demonstrating that expression of miR-10 target genes is downregulated by expression of miR-10a/b (black lines; *n*=11,923, ***P<0.0001, Kolmogorov–Smirnov *Z*-test). (C) A large number of downregulated targets (89) were shared between miR-10a and miR-10b overexpression. Gene ontology (GO) analysis was performed on the shared targets, revealing miR-10 to be involved in functions related to transcription, actin cytoskeleton and ephrin receptor signalling. (D) The expression of the miR-10 target gene *LIN28* was downregulated by overexpression of miR-10a and miR-10b. (E) Both *WNT1* and *MSX1* take part in the midbrain/hindbrain patterning and were downregulated upon overexpression of miR-10a and miR-10b.

MiRNAs have the potential to regulate hundreds of target genes, thus controlling complex gene networks. However, identification of miRNA targets is challenging, due to drawbacks with current *in silico* prediction methods, as well as the apparent cellular context-dependency of miRNAs. In order to identify transcripts that are direct miR-10 targets in human NPCs, we performed an experiment based on a short pulse of miR-10 overexpression, followed by RNA-seq and subsequent bioinformatics analysis (see Fig. 7A for schematic overview of the experiment). To this end, we transduced hESC with the doxycycline-regulated miR-10a and miR-10b lentiviral vectors. Thereafter, cells were differentiated towards a ventral MB fate (which lacks endogenous miR-10 expression) for 11 days. At that time point, doxycycline was added to the culture media to activate mir-10 expression, the cells were harvested 3 days later for RNA isolation and used for poly-A enriched mRNA-sequencing (mRNA-seq). Cells transduced with miR-10 constructs but grown without doxycycline were used as control. The sequencing reads were mapped to the human genome, scale-normalized and subsequently analysed for the expression levels of different transcripts (RefSeq, supplementary material Table S4).

To identify miR-10 target genes in hNPCs, we calculated the fold change for each transcript after miR-10a or miR-10b overexpression, and analysed fold change distributions for transcripts that contain a conserved putative miR-10 target site (TargetScan). We found that both miR-10a and miR-10b overexpression resulted in reduced mRNA levels for the majority of putative miR-10 targets (Fig. 7B), which is in line with the notion that miRNAs predominantly act by reducing mRNA levels (Guo et al., 2010). Thereafter, we selected the putative miR-10 targets that were downregulated by both miR-10a and miR-10b overexpression (Fig. 7C). This list of 89 high-confidence miR-10 targets in hNPCs contained several interesting transcripts, which are likely to influence brain development and neuronal patterning (supplementary material Table S5).

We performed gene ontology analysis on the 89 high-confidence miR-10 targets in hNPCs and found that miR-10 targets were enriched for functions related to transcription, actin cytoskeleton and ephrin receptor signalling (Fig. 7C). One interesting example of the miR-10 targets that were significantly downregulated by both miR-10a and miR-10b overexpression was Lin28A (Fig. 7D), which is an extensively studied RNA-binding protein implicated in brain development and brain cancer (Shyh-Chang and Daley, 2013; Yang et al., 2015).

Finally, we investigated whether the short pulse of miR-10 overexpression also influences genes known to be involved in midbrain/hindbrain patterning. We found that overexpression of either miR-10a or miR-10b led to a robust downregulation of both *WNT1* and *MSX1* (Fig. 7E), which are both expressed in the midbrain/hindbrain boundary and are involved in the patterning of mouse midbrain neurons (Arenas et al., 2015). Taken together, these data identify a large network of genes controlled by miR-10 in hNPCS. Modulation of miR-10 levels in hNPCs is likely to have broad and complex consequences, including influencing midbrain/hindbrain patterning via regulation of WNT1 and MSX1.

## DISCUSSION

The involvement of miRNAs in human brain development has remained largely unexplored, mostly due to the difficulties of obtaining purified populations of neural progenitors of a defined region and the lack of human foetal tissue. However, miRNAs have been shown to be important for brain development and cell type specification in other species. In this study, we provide a comprehensive profiling of miRNA expression in defined populations of regionalized human neural progenitors of a forebrain, midbrain and hindbrain identity. Key to this approach is the use of a differentiation protocol that allows both rostro-caudal and dorso-ventral patterning of hPSC-derived neural progenitors (Kirkeby et al., 2012a), and the use of the CORIN and SOX1 markers to obtain pure populations of cells.

The miRNA-profiling demonstrated that miRNAs display a region-specific expression pattern in hNPCs, suggesting that miRNAs contribute to establishing regional identity in different progenitor populations. The region-specific miRNA expression was confirmed using miRNA array on sub-dissected human foetal brain material. Additionally, the miRNA array demonstrated that several miRNAs are enriched in a stage-specific manner. Thus, microRNAs, like coding genes, appear to be regulated in a spatiotemporal manner during human brain development.

It is interesting that a few miRNAs are strongly dominating the total miRNA expression in hNPCs. In forebrain- and midbrain-patterned cells, miR-92a and miR-92b compose >70% of all miRNAs. Moreover, the miR-92 family was specifically associated with neural progenitors, which suggests that they play a role in regulating human neural progenitor proliferation or specification. Overall, the expression profile of miRNAs in hNPCs is very distinct from the expression profile of miRNAs that have previously been reported for the adult human brain, where miR-92 is expressed at low levels, whereas other miRNA families, such as let-7 and miR-124, dominate the expression profile (see e.g. Boudreau et al., 2014). This difference in miRNA expression between developing and adult brain reinforces the developmental stage dependency of miRNA expression that emerged in our study.

The identification of miR-10 as a miRNA that is highly and specifically expressed in the developing human hindbrain suggested a role for this miRNA in caudalization of human neural progenitors. Both miR-10a and miR-10b are located in the Hox cluster (Woltering and Durston, 2008). Previous experiments in zebrafish have suggested that miR-10 represses its own expression as well as other transcripts in the Hox cluster via a complex autoregulatory loop (Woltering and Durston, 2008). In addition, miR-10 has been linked to differentiation and cancer by regulating multiple target genes (Lund, 2010). Our data show that modulation of miR-10 levels affect a large number of genes, resulting in broad transcriptional changes. The functional analysis in hESCs show that one of the roles for miR-10 in hNPCs is related to patterning, as ectopic expression of miR-10 results in caudalization of these midbrain-patterned hNPCs. The caudalization was evident both by downregulation of fore- and midbrain-specific genes and upregulation of hindbrain-specific genes, while leaving non-regionalized genes unaffected. Thus, expression of miR-10 alone can influence the positional regional identity of human neural progenitors, a phenomenon that appears to be linked to pathways including WNT1 and/or MSX1, as these factors were downregulated by a brief exposure to miR-10 overexpression.

In conclusion, by performing global miRNA expression profiling of defined sets of human neural progenitors, we have established a unique miRNA profile for human neural progenitors from different regions of the brain. Additionally, we have identified several miRNAs associated with pan-neuronal specification and regionalization of human neural progenitors. This comprehensive dataset provides a resource for future studies examining potential functions of miRNAs with distinct expression profiles during human brain development.

## MATERIALS AND METHODS

### BAC recombineering, restriction enzyme analysis and PCR

All reagents for BAC recombineering and the competent *Escherichia coli* (*E. coli*) strain SW102 were obtained from the Biological Resources Branch preclinical repository of the National Cancer Institute (Maryland, USA). A detailed description of the materials is given on the website https://ncifrederick.cancer.gov/research/brb/recombineeringinformation.aspx. The BAC construct containing the genomic locus of SOX1, RP11-426j19, was identified using Genome Browser (UCSC, http://genome.ucsc.edu/) and obtained from Invitrogen. All recombineering experiments were performed according to protocols published previously (Lee et al., 2001; Warming et al., 2005; Yu et al., 2003). The eGFP-pSV40-Neo^R^ reporter cassette, amplification protocols and primers were kindly provided by Dr Yvonne Fischer (Fischer et al., 2010). The proper eGFP-pSV40-Neo^R^ reporter cassette insertion was analysed by restriction analysis with *Age*I and PCR, using primers specific for the overlapping 5′ and 3′ ends: CCGTCTCACTCCGTCTGAATAAAGGCAGGATGATGACCAG; GGGAGGCTAACTGAAACACGTTGCTGATCTCCGAGTTGTG.

### Lentiviral vectors

The vectors used in this study were third-generation SIN vectors. All cloning was performed using standard techniques, and lentiviral vectors were produced as previously described (Zufferey et al., 1997). Vectors were titrated using qRT-PCR analysis, as previously described (Georgievska et al., 2004). The titres of the vectors in this study were 10^9^ TU/ml.

### Human ESC culturing and lentiviral transduction

Human ESC H9 (WA09, passage 31-45) (Thomson et al., 1998) was expanded and maintained on γ-irradiated mouse embryonic fibroblasts (MEFs) in DMEM/F12, 20% KSR, 0.05 mM 2-mercaptoethanol, 0.5% pen/strep, 0.5% glutamate and 10 ng/ml FGF-2 (R&D Systems). The cells were passaged once weekly with dispase (5 mg/ml, Stemcell Technologies) or EDTA (0.5 mM).

At the time of lentiviral transduction the ESCs were plated onto Matrigel (1:40, BD Biosciences) without MEFs and infected at an MOI of 50. The infected hESCs were maintained on Matrigel for 3 days before plated back onto MEFs for an additional 7 days of expansion prior to differentiation.

### Differentiation into forebrain-, midbrain- and hindbrain-like cells

Differentiation was initiated by detaching the hESC colonies with dispase or EDTA and grown as free-floating aggregates in DMEM/F12:Neurobasal (1:1) supplemented with N2 (1:100), B27 (without vitamin A) (1:50) for 4 days and Y-27632 (10 mM, Tocris Bioscience) for the initial 2 days. The formed EBs were thereafter plated in DMEM/F12:Neurobasal (1:1), N2 (1:200) and B27 (without vitamin A) (1:100) onto a surface coated with polyornithine (PO), fibronectin (FN) and laminin (lam). From d0 to d9, SB431542 (10 mM, Tocris Bioscience) and noggin (200 ng/ml, R&D) were present in the medium for neuralization, as well as SHH-C24II (R&D, 200 ng/ml) and CT99021 (Axon Medchem) (0.6-0.8 μM for MB, 2 μM for HB and 3 μM for posterior HB) for patterning along the dorsal-ventral axis and the anterior-posterior axis, respectively. For strong ventralization the medium was supplemented with Purmorphamine (Stemgent, 0.5 μM). At day 11, the attached cell clusters were dissociated to a single-cell suspension with accutase, and were replated in 20 μl droplets of 10,000-15,000 cells/μl onto dry PO/FN/lam-coated plates in Neurobasal, B27 (without vitamin A) (1:50), brain-derived neurotrophic factor (BDNF) (20 ng/ml), glial-derived neurotrophic factor (GDNF) (10 ng/ml) and ascorbic acid (200 mM). When differentiating hESCs transduced with LV.GFP, LV-miR-10a or LV.miR10b, the transgenes were turned on by adding doxyxcycline (2 μg/ml, Saveen & Werner) to the media. All groups were performed in triplicates. For terminal differentiation cAMP (0.5 mM) and DAPT (1 mM) were added to the medium from day 14 onwards.

### Differentiation into mesendoderm

Prior to differentiation, the human ESCs were grown on MEFs until confluency. Medium was thereafter changed to RPMI (Mediatech) supplemented with 0.5% pen/strep, 0.5% glutamate and 100 ng/ml activin A (R&D) for one day. The second day and onwards the medium was further supplemented with foetal bovine serum (FBS) (Gibco).

### Nucleofection and karyotyping

Prior to nucleofection (Placantonakis et al., 2009), hESCs were cultured on matrigel (BD Biosciences) in MEF-conditioned human ESC medium. Cells were then dissociated with accutase and re-suspended in solution V (Amaxa) at a density of 5×10^6^ cells/100 μl. The cell suspension was mixed with 5 μg freshly prepared (Big BAC DNA kit, Princeton Separations) and nucleofected under protocol B-16 (Amaxa). Freshly nucleofected cells were thereafter plated on MEFs in human ESC medium supplemented with 10 μM Y-27632 at a density of 3.3×10^4^ cells/cm^2^. Y-27632 was added to the medium for the first 2 days after nucleofection. Selection with G418 started on day 4 at 12.5 μg/ml, followed by 25 μg/ml on day 14. After 2-3 weeks of G418 selection colonies were isolated and expanded. The *SOX1*-GFP^+^ clone was analysed cytogenetically based on 20 G-banded metaphases by Cell Line Genetics, Madison, USA.

### Quantitative RT-PCR

Total RNA was isolated using the RNeasy Micro Kit (Qiagen) and cDNA was created by reverse transcription with random hexamer primers and Superscript III (Invitrogen). miRNA was isolated using the miRNeasy Kit (Qiagen), followed by Universal cDNA synthesis kit (Exiqon). LNA PCR primer sets, hsa-miR-10a, hsa-miR-10b, hsa-miR-124-5p and hsa-miR-103 (the latter two were used as normalization miRNA) were purchased from Exiqon.

The cDNA was analysed by quantitative PCR with SYBR Green I master (Roche) on a LightCycler 480 (Roche). All samples were run in technical triplicates, and the average Ct-values were used for calculations. Data are represented using the ΔΔCt method normalized to the housekeeping gene *ACTB*. Error bars on graphs represent variation from biological and/or technical replicates. Human-specific primers are listed in supplementary material Table S6.

Differences between groups were analysed using one-way ANOVA with Bonferroni post-hoc test.

### FACS and analysis

All flow cytometry for isolation and analysis were run on a FACSAria and Accuri, respectively (BD Biosciences). The cells were dissociated with accutase for 30 min at 37°C with intermediate triturations, washed in HBSS supplemented with 5% foetal bovine serum (FBS) and centrifuged at 300 ***g*** for 5 min. The cells were re-suspended at a concentration of 10 M cells/ml and filtered (70 μm pore size, BD Bioscience) to remove larger aggregates. To visualize CORIN expression, a human-specific rat anti-CORIN antibody (supplementary material Table S4) was added to the cell suspension and incubated for 30 min on ice. Cells were then washed and incubated with a fluorochrome-conjugated secondary antibody (anti-rat APC, 1:400, eBioscience ref17-4822-82) for 30 min on ice in the dark. The labelled cells were sorted for live populations, excluding dead cells, by 7AAD (BD Bioscience).

### Human and rodent tissue collection

Human tissue was obtained from legally aborted embryos, with approval of the Swedish National Board of Health and Welfare. The VMs were collected from post conception (P.C.) week 5-11. Mice and rats were maintained and handled according to the guidelines set by the Ethical Committee for the use of animals at Lund University. For staging of rodent embryos, the morning of the vaginal plug was considered as embryonic day 0.5 (E0.5).

### Immunofluorescence and microscopy

Prior to immunofluorescence the cells and tissue were fixed in 4% paraformaldehyde. The primary antibodies (supplementary material Table S7) were diluted in blocking solution and incubated at 4°C overnight, followed by a 2 h incubation with a fluorophore-conjugated secondary antibody (Molecular Probes or Jackson Laboratories, 1:400) at room temperature. For imaging and analysis we used a confocal microscope (Leica) or inverted microscope (Leica, DFC360 FX-DMI 6000B).

### sRNA sequencing and analysis

All samples, groups of duplicates to quadruplicates, were prepped with miRNeasy Kit (Qiagen) and sent to SciLife in Uppsala, Sweden, for RNA sequencing using the Illumina HiSeq 2000 Sequencing System. Sequencing data were processed with fastx (http://hannonlab.cshl.edu/fastx_toolkit/index.html), and adapter sequences and sequences shorter than 15 nucleotides were removed. Data were thereafter aligned (Bowtie2) to known human mature and hairpin microRNAs downloaded from miRBase 2013-06-22 (mirbase.org), and identical reads were counted. Prior to final analysis, the dataset was normalized, so that every sample had the same total counts. Final analysis and selection of miRNAs were performed in Excel (Microsoft Office). The RNA-seq data have been deposited in the NCBI Gene Expression Omnibus and are accessible through GEO series accession number GSE68189.

### miRNA microarray

miRNA from the human embryonic tissue was isolated using the miRNeasy Kit (Qiagen) and sent to Exiqon for a miRCURY LNA Universal RT microRNA PCR; the microarray contained the 59 strongest-expressed miRNA from the RNA sequencing. The data were normalized to the average expression levels of all miRNAs in all samples, as this was found to be the most stable normalizer. In order to compare the groups of different time points in 5E, a one-way ANOVA was used.

### mRNA sequencing and analysis

Total RNA was extracted using the RNeasy Mini Kit, following instructions of the supplier (Qiagen). A total of 12 samples (three replicates per group) were used for mRNA sequencing. cDNA libraries of mRNA samples were prepared using the NuGEN Ovation RNA-Seq System, including poly-A enrichment, and sequenced using Illumina HiSeq 2000. The 50-bp single-end reads were mapped to the human genome (hg19) and visualized in the UCSC genome browser. Reads were quantified to Refseq. Differentially expressed genes were calculated in Microsoft Excel after scaling all data to the total number of reads in each sample.

Gene ontology analysis was conducted using the online DAVID bioinformatics database tool (http://david.abcc.ncifcrf.gov). Genes with a read number >10 in the mRNA-seq control hNPCs were used in a background list for the functional annotation analysis of each dataset. Using medium default stringency, we identified all significantly enriched (*P*<0.05) Gene Ontology (GO) biological processes (BP) and Kyoto Encyclopaedia of Genes and Genomes (KEGG) pathways in a functional annotation chart. KEGG pathways were labelled in bold font. For the identification of computationally predicted miRNA targets, TargetScanHuman 6.2 was used (http://www.targetscan.org/vert_61/). The RNAseq data have been deposited in the NCBI Gene Expression Omnibus and are accessible through GEO series accession number GSE71204.

## Supplementary Material

Supplementary information
