# Supplementary material for: Comprehensive analysis of microRNA expression in regionalized human neural progenitor cells reveals microRNA-10 as a caudalizing factor
Source: Development. 2015 Sep 15;142(18):3166–77. doi: 10.1242/dev.122747 (PMC4582174; doi:10.1242/dev.122747)
Supplement: Supplementary information [file supp_142_18_3166__index.html]

Supplementary information 

# Comprehensive analysis of microRNA expression in regionalized human neural progenitor cells reveals microRNA-10 as a caudalizing factor

## DEV122747 Supplementary information

- Supplementary information
