# Supplementary material for: Comprehensive analysis of microRNA expression in regionalized human neural progenitor cells reveals microRNA-10 as a caudalizing factor
Source: Development. 2015 Sep 15;142(18):3166–77. doi: 10.1242/dev.122747 (PMC4582174; doi:10.1242/dev.122747)
Supplement: Supplementary information [file supp_142.18.3166_DEV122747supp.pdf]

SUPPLEMENTARY FIGURES

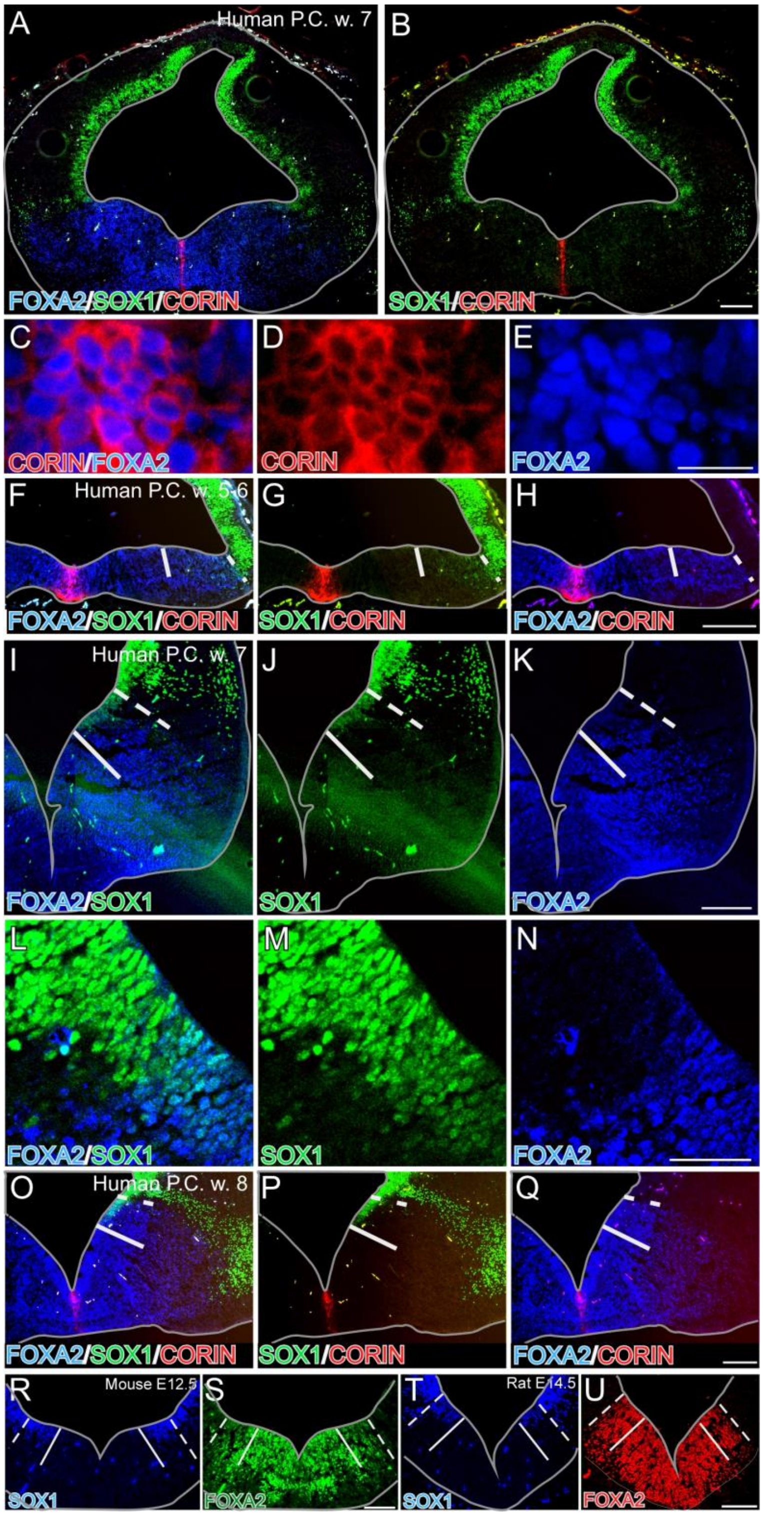

Figure S1. The human midbrain expresses SOX1 and CORIN

SOX1 positive cells were found situated in the human mesencephalon within the ventricular zone with two exceptions, the roof plate and the FOXA2 positive floor plate (FP) (A-B).

Development • Supplementary information

CORIN was shown to solely be restricted to the FP cells in the midline (A-B, F-H, O-Q) and were shown to co-express FOXA2 (C-E). At week 5-6 p.c., SOX1 positive cells were found within the lateral FOXA2 domain, between the dashed and solid marking lines, although the level of expression appeared lower compared to the more lateral SOX1 positive domain (F-H). This difference in SOX1 expression seemed to increase over time and at p.c. week 7 to 8, no difference could be observed (I-N, O-Q). The lateral FOXA2/SOX1 double positive domain was also found in mice and rats (R-U). Scale bars: A-B, F-K, O-Q, 200 µm; C-E; 20 µm; L-N, R-U; 100 µm.

Development • Supplementary information

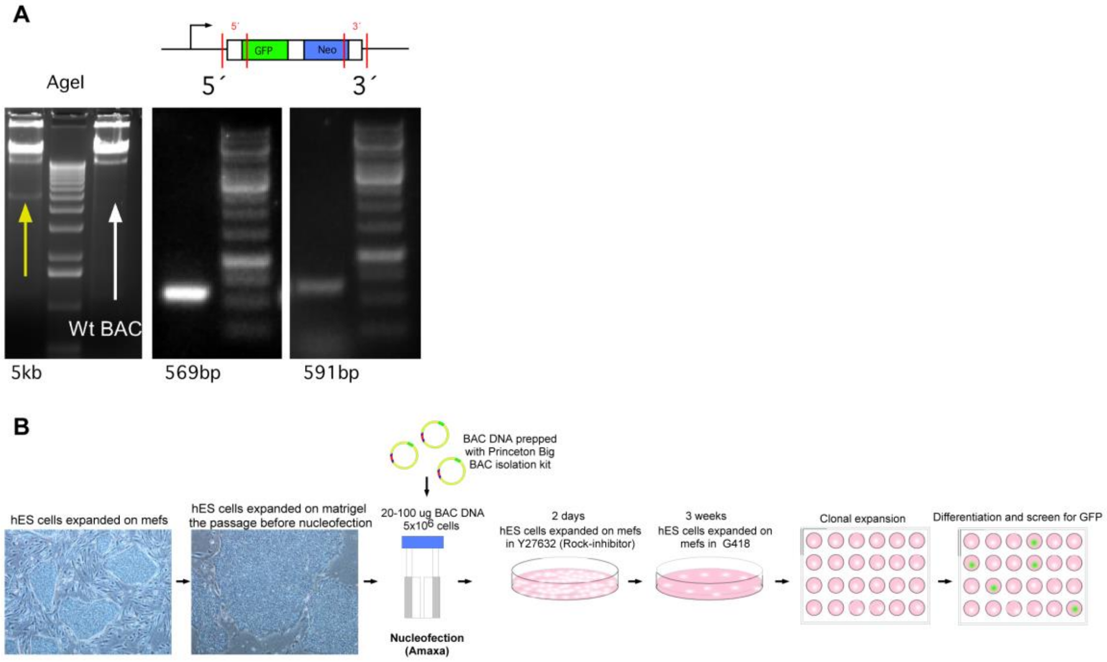

Figure S2. Validation of the human SOX1-GFP construct and generation of a BAC reporter cell line. (A) Proper eGFP-pSV40-Neo^R reporter cassette insertion was validated with restriction enzyme and PCR analysis. (B) The BAC based SOX1-GFP reporter construct was introduced into the hESCs by nucleofection, followed by clonal expansion and screening during differentiation. Scale bar: 100 µm.

Development • Supplementary information

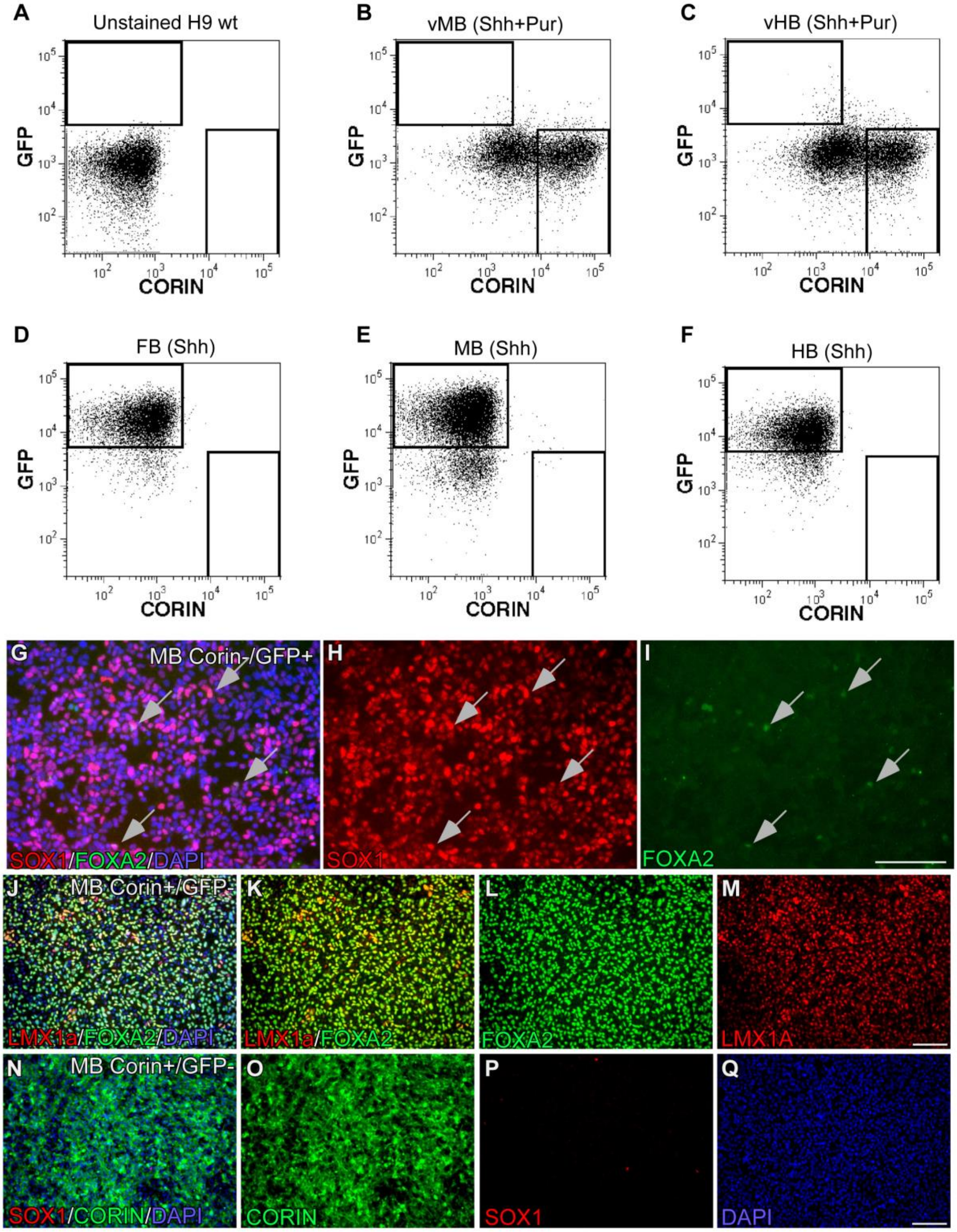

Figure S3. The *SOX1*-GFP reporter hESC line can readily differentiate into MB NE and FP cells

(A-C) When adding the SHH agonist Purmorphamine to FB, MB and HB differentiation cultures from day 0-9 the cells were effectively ventralized, as the majority of the cells were

Development • Supplementary information

GFP negative at day 14, and MB and HB FP cells started to express high levels of the FP marker CORIN. (D-F) This is in contrast to differentiation without Purmorphamine, where the majority of cells was GFP+ at day 14 and had a very low expression of CORIN. (G-I) Isolated GFP+/CORIN- MB NE populations were shown to highly express SOX1 with only a few cells being positive for the FP marker FOXA2 and the majority of those being co-labeled with SOX1 (arrows). (J-M) In contrast, the GFP-/CORIN+ FP cells were rich in FOXA2 expressing cells and in the MB condition FOXA2 was efficiently co-labeled with LMX1A. (N-Q) The isolated GFP-/CORIN+ populations were negative for SOX1.

Development • Supplementary information

Table S1

Click here to Download Table S1

Table S2

Click here to Download Table S2

Table S3

Click here to Download Table S3

Table S4

Click here to Download Table S4

Table S5

Click here to Download Table S5

Table S6. Human specific primers

Corin	F	CATATCTCCATCGCCTCAGTTG
	R	GGCAGGAGTCCATGACTGT
FoxA2	F	CCGTTCTCCATCAACAACCT
	R	GGGGTAGTGCATCACCTGTT
FoxG1	F	TGGCCCATGTCGCCCTTCCT
	R	GCCGACGTGGTGCCGTTGTA
Gbx2	F	GTTCCCGCCGTCGCTGATGAT
	R	GCCGGTGTAGACGAAATGGCCG
GFP	F	CTGCTGCCCGACAACCAC
	R	ACCATGTGATCGCGCTTCTC
HoxA2	F	CGTCGCTCGCTGAGTGCCTG
	R	TGTCGAGTGTGAAAGCGTCGAGG
HoxA4	F	ACGCTCTGTTTGTCTGAGCGCC
	R	AGAGGCCGAGGCCGAATTGGA
Lhx1	F	AGGTGAAACACTTTGCTCCG
	R	CTCCAGGGAAGGCAAACTCT
Lmx1a	F	CGCATCGTTTCTTCTCCTCT
	R	CAGACAGACTTGGGGCTCAC
Lmx1b	F	CTTAACCAGCCTCAGCGACT
	R	TCAGGAGGCGAAGTAGGAAC
Otx2	F	ACAAGTGGCCAATTCACTCC
	R	GAGGTGGACAAGGGATCTGA
Pax6	F	TGGTATTCTCTCCCCCTCCT
	R	TAAGGATGTTGAACGGGCAG
Shh	F	CCAATTACAACCCCGACATC
	R	AGTTTCACTCCTGGCCACTG
Sox1	F	GGGAAAACGGGCAAAATAAT
	R	TTTTGCGTTCACATCGGTTA
Sox17	F	CCAGACCGCGACAGGCCAGAAC
	R	AGTGAGGCACTGAGATGCCCCGAG

Table S7. List of antibodies

Species	Antibody	Dilution	Company	Order no.
goat	Foxa2	1:600	Santa Cruz	sc-6554
mouse	Map2	1:500	Sigma	M1406
rabbit	Lmx1a	1:2000	Millipore	AB10533
mouse	Oct3/4	1:500	Chemicon	AB9558
rat	Corin	1:200	R&D Systems	MAB2209
goat	Otx2	1:2000	Neuromic	GT15095
goat	Sox17	1:100	R&D Systems	MAB1924
rabbit	Brachury	1:200	Abcam	ab20680
rb	Sox1	1:200	Cellsignaling	41945
chicken	GFP	1:1000	Abcam	ab13970
